# 

**Published:** 1866-05

**Authors:** 


### CONTENTS.

ORIGINAL COMMUNICATIONS.

Dental Materia Medica ... 193
Dental Ethics ... 197
Dental Hobbies ... 205
Extracting Teeth ... 210
Little Things ... 218
More Light ... 220

PROCEEDINGS OF SOCIETIES.

Synopsis of Discussions, Missouri State Dental Society ... 223
Minutes of Missouri State Dental Society ... 230

EDITORIAL.

Acknowledgment ... 235
Nashville Dental Society ... 236
Central Ohio Dental Association ... 237
Central States Dental Association ... 237
Mississippi Valley Association ... 238

